# Chinese Herbal Medicine Meets Biological Networks of Complex Diseases: A Computational Perspective

**DOI:** 10.1155/2017/7198645

**Published:** 2017-06-11

**Authors:** Shuo Gu, Jianfeng Pei

**Affiliations:** ^1^Center for Quantitative Biology, Academy for Advanced Interdisciplinary Studies, Peking University, Beijing 100871, China; ^2^Institute for Medical Engineering and Science, Massachusetts Institute of Technology, Cambridge, MA 02139-4307, USA

## Abstract

With the rapid development of cheminformatics, computational biology, and systems biology, great progress has been made recently in the computational research of Chinese herbal medicine with in-depth understanding towards pharmacognosy. This paper summarized these studies in the aspects of computational methods, traditional Chinese medicine (TCM) compound databases, and TCM network pharmacology. Furthermore, we chose arachidonic acid metabolic network as a case study to demonstrate the regulatory function of herbal medicine in the treatment of inflammation at network level. Finally, a computational workflow for the network-based TCM study, derived from our previous successful applications, was proposed.

## 1. Introduction

The past century has witnessed tremendous achievements in orthodox medicine and pharmaceutical science as thousands of drugs were created to save human lives throughout the world. Meanwhile, traditional Chinese medicine (TCM), one of the major alternative medicines, actively embraces science and technology, resulting in fruitful clinical outcomes [1]. As an essential part of TCM, Chinese herbal medicine plays an important role in treating patients under the framework of TCM theory, which is characterized by being holistic, systematic, and individualistic, while it is nowadays echoed by the topics of personalized and precise medicine [2–4].

Chinese herbal medicine, originated from the first literature* Shennong's Materia Medica* (~220 CE), has accumulated an abundance of healing knowledge in both theory and practice through thousands of years. To be specific, medical materials are classified according to four natures (hot, warm, cool, and cold), five flavours (acrid, sweet, bitter, sour, and salty), and different meridians, which require careful combination in a formula for each individual patient [5]. Here also the issue of medical materials' compatibility is raised, which further determines the four major components in a formula: principal, associate, assistant, and coordinator [6]. Enriched by tremendous practical data, formulas are developing and evolving in the history with better clinical performance.

To better utilize the hidden wisdom in Chinese herbal medicine, one needs to interpret its property and function using analytical tools well developed in modern science [7]. Such methods include but are not limited to analytical chemistry, molecular pharmacology, animal model, and computational method [8–10]. In this paper, we will discuss from the computational perspective the topic of Chinese herbal medicine addressing the biological networks of complex diseases and try to propose a computational workflow derived from our previous research [11–13].

## 2. Computational Method

**Figure 1:**
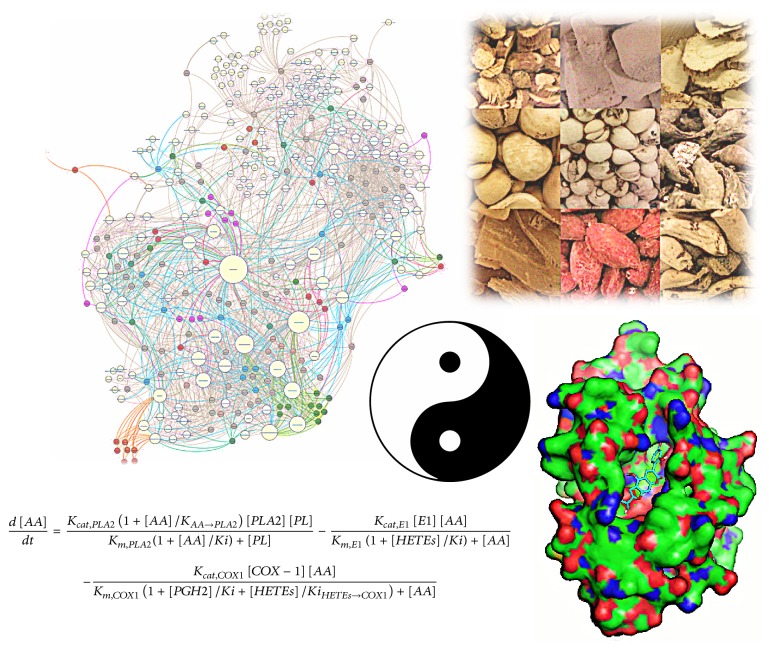
An illustration of Chinese herbal medicine and some computational methods. At the centre is a yin-yang symbol representing the philosophy of traditional Chinese medicine, surrounded by a picture of herbal materials, a protein-ligand complex derived from docking, a graph of herbal medicine and associated pathways, and an example of ordinary differential equation.

Generally speaking, the computational methods applied in herbal medicine are largely associated with computational chemistry and computational biology because these two disciplines provide researchers with intermediate tools to process chemicals from herbs and targets in pathways. For example, given a small molecule discovered from a herb of interests, one can use chemical similarity searching to find compounds with similar structure and presumably similar bioactivity [14]. Furthermore, docking is a powerful method to quantify the binding energy of a ligand to its target [15]. Based on this method, scientists have developed a web server to identify potential drug targets from multiple therapeutic areas for natural compounds in herbal medicine, which is also known as reverse docking [16–18]. Molecular dynamics is another widely used method to simulate the interaction of a small molecule and a macromolecule, shedding light on the binding kinetics of a biological system at atomic level [19, 20]. For example, Chang et al. integrated this method with docking in the screening of a TCM database against hemagglutinin, a target in influenza virus [21]. On the other hand, we can apply systems biology to study the behaviour of a biological network with and without the treatment of a certain herb in order to simulate the efficacy and side effects of the herb at network level [11, 22]. For example, given the topology and its related parameters, a set of ordinary differential equations (ODEs) can faithfully describe the dynamic property of the network with the perturbation of herbal medicine [11, 23]. However, since ODEs are highly dependent on detailed biological pathways, lack of either topology or parameter information would result in failure of this method. In this case, Boolean network modelling could be a useful alternative [24]. In conclusion, all of these methods are developed to study a biological system (e.g., herb and cell) at certain levels while the combination of them can cover a wide range of scales on both time and space (Figure 1).

## 3. TCM Compound Database

The major goal of TCM research is to understand the mechanisms of herb-human interplay, which in one modern way that can be translated into the interaction of chemical components and macromolecular targets at molecular level. With the help of cheminformatics and data science, several TCM compound databases of high qualities were curated: Traditional Chinese Medicine Database [25], Chinese Traditional Medicinal Herbs Database [26], TCM Database@Taiwan [27], Traditional Chinese Medicine Systems Pharmacology Database [28], Traditional Chinese Medicines Integrated Database [29], and Herbal Ingredients' Targets Database [30].

Here are some highlights of several essential TCM databases. Traditional Chinese Medicine Database is one of the pioneers in this field. It offers detailed information for more than 20000 TCM natural compounds, especially for the annotation of related experiments and references [25]. TCM Database@Taiwan claims to be the largest TCM database containing more than 30000 compounds from 352 TCM ingredients. These ingredients were organized in different categories according to the TCM theory, while the molecules under each ingredient were curated in both cdx (2D) and mol2 (3D) formats [27]. Traditional Chinese Medicine Systems Pharmacology Database aims to be a full-stack assistant in computational TCM research, offering not only 3D molecular structures but also physicochemical and pharmacokinetic properties of 12144 compounds. In addition, it provides 3311 potential drug targets and user-tailored networks to show the interaction of drug-target-disease [28].

While most of the researches focus on virtual screening of the databases by various computational tools like chemical similarity search, docking, and molecular dynamics [31–34], drug-likeness analysis was also applied to the TCM databases in order to investigate the pharmaceutical potential of natural compounds. For example, Shen et al. published a series of papers on the topic of drug-likeness analysis of traditional Chinese medicines by comparing the molecular properties and scaffold architectures for drug-like compounds, non-drug-like compounds, and natural compounds from traditional Chinese medicines. Subsequently, they applied machine learning approaches on a TCM database, identifying almost 60% of the molecules as drug-like [35–37]. Furthermore, Xue et al. developed a method with the combination of network topology analysis and cheminformatics measurements to predict the safety for natural compounds of an in-house TCM database. With that, they were able to discover that a promising lead compound Silibinin was surprisingly very similar to a withdrawn drug called Plicamycin [38].

## 4. TCM Network Pharmacology

The concept of network pharmacology was proposed by Hopkins in 2008 [39], although the idea of multitarget drugs in a network approach has been prevailing since an earlier date [40]. Because the previous paradigm (one gene, one drug, one disease) in drug discovery no longer satisfied the treatment of complex diseases like AIDS, cancer, and neurological disorders, scientists realized the necessity to shift this paradigm into a network-targeted combination therapy with robust efficacy and low toxicity [39, 41].

In the field of TCM network pharmacology, people have begun to address essential questions by utilizing various computational methods. For example, Li et al. proposed a TCM research framework as “phenotype network, biological network, herb network”, with which they developed several useful tools to elucidate the network pharmacology alongside the systematic interpretation of TCM theory and practice [42–45]. On the other hand, using network analysis and molecular docking, Gu et al. introduced multiple computational approaches in the research of herbal pharmacology and efficacy evaluation [46, 47]. Other novel studies in both methods and applications have been accumulating recently with rapid advances in the understanding of herbal medicine [48–54].

Among the studies mentioned above, Zhao and Li developed a systematic approach focusing on three parts of TCM network pharmacology. The first one was drug target identification by relating pharmacological and genomic spaces. The computational framework drugCIPHER was used to infer drug-target interactions in a genome-wide scale [43]. The second one was building on the research of drug-gene-disease relationship. They applied a comodule approach to elucidate drug-disease associations, which was automated by the software called comCIPHER [45]. The third part included several methods that contribute to the analysis of TCM combination in the biological network scenario: for example, the Distance-Based Mutual Information Model (DMIM) to uncover the combination rule of TCM formulas and Network Target Based Identification of Multicomponent Synergy (NIMS) to screen synergistic TCM drug combination [55, 56].

## 5. TCM Interacting with Biological Networks of Complex Diseases

For the last decade, we have been exploring network-based drug design to address complex diseases like inflammation, HIV, and cancer [57–59]. In order to shift a biological network from a disease state to the normal state, we developed a Multitarget Optimum Intervention (MTOI) method to identify key targets in a network for intervention [60, 61]. Here, the arachidonic acid (AA) metabolic network is taken to illustrate our strategy.

**Figure 2:**
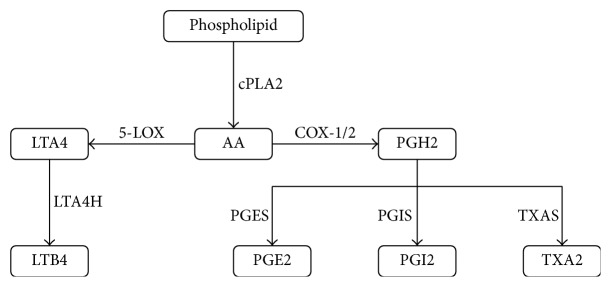
The basic structure of the arachidonic acid metabolic network associated with inflammatory processes. When phospholipid is catalyzed by cPLA2 into arachidonic acid, there are two major pathways (5-LOX and COX-1/2) in this network. Each circled product is catalyzed by the cognate protein nearby while the arrow indicates the metabolic direction.

The AA metabolic network contains two major pathways in inflammatory processes with five key enzymes: cyclooxygenase-1/2 (COX1/2), 5-lipoxygenase (5LOX), microsomal prostaglandin E synthase-1 (PGES), and leukotriene A4 hydrolase (LTA4H). Overproduction of two metabolites prostaglandin E2 (PGE2) and leukotriene B4 (LTB4) leads to inflammatory syndromes; for example, PGE2 is highly associated with arthritis while LTB4 is associated with coughs and asthma. Furthermore, the concentration ratio of prostacyclin (PGI2) and thromboxane A2 (TXA2) is linked to drugs' side effects (Figure 2) [60, 61]. With a dynamic model of 26 ODEs, we faithfully described its metabolic process and successfully screened several multitarget inhibitors [62–64].

Using a combined approach of molecular docking and network simulation, we were able to reveal the regulatory roles of herbal medicine in AA metabolic network [11]. Firstly, we collected 28 anti-inflammatory herbs and selected all the available molecules from Traditional Chinese Medicine Database under some criteria. The structures of key enzymes in AA network were either obtained from Protein Data Bank (PDB) or modelled. Then, we used AutoDock to investigate the binding energy in each complex among 5 proteins and 237 ligands. The docking results were further imported in the network simulation described by 26 ODEs. Finally, the reduction of inflammatory mediators (PGE2 and LTB4) and the ratio of [PGI2]/[TXA2] as the efficacy and side effects, respectively, were calculated for each herb and formula in the treatment of inflammation.

The network output provided us with the molecular interpretation of the regulatory function of each herb or formula. Concretely, we found that herbs decreased LTB4 production more than PGE2 production, while most nonsteroidal anti-inflammatory drugs (NSAIDs) mainly reduce the production of PGE2. In addition, [PGI2]/[TXA2] ratio of each herb indicated mild side effects. From the simulation of herbal formula, we discovered an interesting phenomenon that herbs in a formula tended to inhibit different enzymes in this network, thus covering most targets to outperform a single herb. Moreover, our method helped to understand the dosage effect of a formula, which could reach the same therapeutic effect at a low dose rather than an individual herb at a high dose. Finally, the simulation results enabled us to design several new herbal combinations for better PGE2 reduction, which was not achieved in the previous collection of herbs.

## 6. A Computational Workflow for Network-Based TCM Study

**Figure 3:**
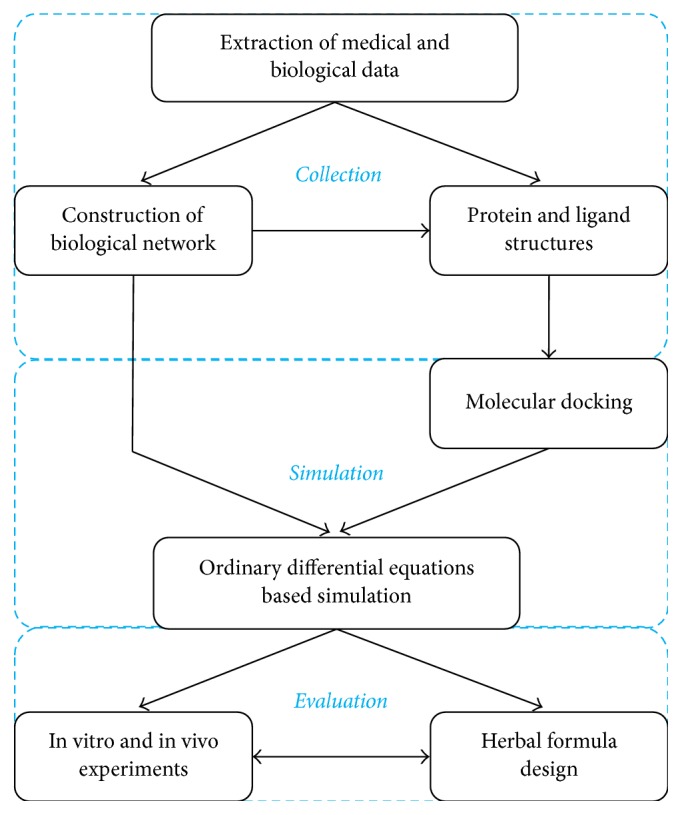
A computational workflow for network-based TCM study. This workflow has three parts in general. The part of collection includes data extraction from various sources, chemical and biological structures from databases, and the construction of biological network. In the simulation part, different methods like docking and ODE-based simulation are applied to study the interaction of herb and biological network. Finally, in the evaluation part, we use wet experiments to verify the computational prediction and iteratively design new formulas based on dry and wet experiments.

On the basis of our previous applications, here we would like to propose a computational workflow for herbal medicine study (Figure 3). In this workflow, we start a project from extracting medical and biological data computationally or manually. These data are usually from various resources like books, papers, and clinical reports. As one of the building blocks in computational herbal medicine, construction of biological networks associated with diseases is vital and inevitable. Fortunately, Kyoto Encyclopedia of Genes and Genomes (KEGG) provides us with a fundamental framework consisting of many essential biological networks [65]. Nevertheless, literature survey is still necessary before one constructs a detailed network with sufficient kinetic parameters. With enough information obtained, we can move on to collect the compounds from TCM databases and the protein structures from PDB. For those proteins without experimental data available, methods like homology modelling can be used to build the 3D structure. In order to quantify the interactions between compounds and proteins, we may apply docking or other methods like molecular dynamics. The predicted dissociation constant of each protein-ligand complex will be imported into the ODE-based simulation to model the dynamics of the biological network. With the outputted data, the efficacy and toxicity of a herb or a formula could be interpreted at a system level. Last but not least, based on the simulation and further validations from wet experiments, one can design new TCM formulas for better performance.

## 7. Conclusions

Computational studies on Chinese herbal medicine in the scenario of biological networks associated with diseases are a new yet promising direction, because they enable researchers to investigate the pharmacological mechanism of TCM at the molecular and network levels, which in return illuminate scientists to reuse the ancient medical knowledge in a systematic and predictable manner. Although not mature in its initial stage, this field has already gained fruitful outcomes in theory, shedding light on the rigorous applications of herbal medicine and a new paradigm of drug discovery. As a foundation of computational herbal medicine, the interplay of TCM database and biological network generated numerous insightful results during the past decade, providing us with a modern understanding of this complex system. Furthermore, our proposed workflow, including many cutting-edge computational techniques, aims to offer a novel approach to elucidate herbal medicine in silico, providing guides for the in vitro and in vivo experiments.

## References

[1] Normile D. (2003). The new face of traditional Chinese medicine. *Science*.

[2] Wang M., Lamers R.-J. A. N., Korthout H. A. A. J. (2005). Metabolomics in the context of systems biology: bridging traditional Chinese medicine and molecular pharmacology. *Phytotherapy Research*.

[3] Zhang A., Sun H., Wang P., Han Y., Wang X. (2012). Future perspectives of personalized medicine in traditional Chinese medicine: a systems biology approach. *Complementary Therapies in Medicine*.

[4] Kitano H. (2002). Computational systems biology. *Nature*.

[5] Li Z., Xu C. (2011). The fundamental theory of traditional Chinese medicine and the consideration in its research strategy. *Frontiers of Medicine in China*.

[6] Jia W., Gao W.-Y., Yan Y.-Q. (2004). The rediscovery of ancient Chinese herbal formulas. *Phytotherapy Research*.

[7] Jiang W.-Y. (2005). Therapeutic wisdom in traditional Chinese medicine: a perspective from modern science. *Trends in Pharmacological Sciences*.

[8] Zhao J., Jiang P., Zhang W. (2009). Molecular networks for the study of TCM pharmacology. *Briefings in Bioinformatics*.

[9] Li W.-F., Jiang J.-G., Chen J. (2008). Chinese Medicine and Its Modernization Demands. *Archives of Medical Research*.

[10] Lukman S., He Y., Hui S.-C. (2007). Computational methods for traditional chinese medicine: a survey. *Computer Methods and Programs in Biomedicine*.

[11] Gu S., Yin N., Pei J., Lai L. (2013). Understanding traditional Chinese medicine anti-inflammatory herbal formulae by simulating their regulatory functions in the human arachidonic acid metabolic network. *Molecular BioSystems*.

[12] Gu S., Yin N., Pei J., Lai L. (2013). Understanding molecular mechanisms of traditional Chinese medicine for the treatment of influenza viruses infection by computational approaches. *Molecular BioSystems*.

[13] Lu L., Meehan M. J., Gu S. (2015). Mechanism of action of thalassospiramides, a new class of calpain inhibitors. *Scientific Reports*.

[14] Willett P., Barnard J. M., Downs G. M. (1998). Chemical similarity searching. *Journal of Chemical Information and Computer Sciences*.

[15] Gschwend D. A., Good A. C., Kuntz I. D. (1996). Molecular docking towards drug discovery. *Journal of Molecular Recognition*.

[16] Li H., Gao Z., Kang L. (2006). TarFisDock: A web server for identifying drug targets with docking approach. *Nucleic Acids Research*.

[17] Cai J., Han C., Hu T. (2006). Peptide deformylase is a potential target for anti-Helicobacter pylori drugs: Reverse docking, enzymatic assay, and X-ray crystallography validation. *Protein Science*.

[18] Zhang S., Lu W., Liu X. (2011). Fast and effective identification of the bioactive compounds and their targets from medicinal plants via computational chemical biology approach. *Medicinal Chemistry Communication*.

[19] Durrant J. D., McCammon J. A. (2011). Molecular dynamics simulations and drug discovery. *BMC Biology*.

[20] Gu S., Silva D.-A., Meng L., Yue A., Huang X. (2014). Quantitatively Characterizing the Ligand Binding Mechanisms of Choline Binding Protein Using Markov State Model Analysis. *PLoS Computational Biology*.

[21] Chang T.-T., Sun M.-F., Chen H.-Y., Tsai F.-J., Chen C. Y.-C. (2011). Drug design for hemagglutinin: Screening and molecular dynamics from traditional Chinese medicine database. *Journal of the Taiwan Institute of Chemical Engineers*.

[22] Butcher E. C., Berg E. L., Kunkel E. J. (2004). Systems biology in drug discovery. *Nature Biotechnology*.

[23] Gennemark P., Wedelin D. (2007). Efficient algorithms for ordinary differential equation model identification of biological systems. *IET Systems Biology*.

[24] Wang R.-S., Saadatpour A., Albert R. (2012). Boolean modeling in systems biology: an overview of methodology and applications. *Physical Biology*.

[25] He M., Yan X., Zhou J., Xie G. (2001). Traditional Chinese medicine database and application on the web. *Journal of Chemical Information and Computer Sciences*.

[26] Qiao X., Hou T., Zhang W., Guo S., Xu X. (2002). ChemInform Abstract: a 3D Structure Database of Components from Chinese Traditional Medicinal Herbs. *ChemInform*.

[27] Chen C. Y. (2011). TCM Database@Taiwan: the world's largest traditional Chinese medicine database for drug screening *in silico*. *PLoS ONE*.

[28] Ru J., Li P., Wang J. (2014). TCMSP: a database of systems pharmacology for drug discovery from herbal medicines. *Journal of Cheminformatics*.

[29] Xue R., Fang Z., Zhang M., Yi Z., Wen C., Shi T. (2013). TCMID: Traditional Chinese medicine integrative database for herb molecular mechanism analysis. *Nucleic Acids Research*.

[30] Ye H., Ye L., Kang H. (2011). HIT: Linking herbal active ingredients to targets. *Nucleic Acids Research*.

[31] Zhao M., Zhou Q., Ma W., Wei D.-Q. (2013). Exploring the ligand-protein networks in traditional chinese medicine: current databases, methods, and applications. *Evidence-Based Complementary and Alternative Medicine*.

[32] Wang L., Zhang S., Zhu J. (2014). Identification of diverse natural products as falcipain-2 inhibitors through structure-based virtual screening. *Bioorganic and Medicinal Chemistry Letters*.

[33] Fu A. K. Y., Hung K.-W., Huang H. (2014). Blockade of EphA4 signaling ameliorates hippocampal synaptic dysfunctions in mouse models of Alzheimer's disease. *Proceedings of the National Academy of Sciences of the United States of America*.

[34] Chang S. S., Huang H. J., Chen C. Y. C. (2011). High performance screening, structural and molecular dynamics analysis to identify H1 inhibitors from TCM Database@Taiwan. *Molecular BioSystems*.

[35] Shen M., Tian S., Li Y. (2012). Drug-likeness analysis of traditional Chinese medicines: 1. property distributions of drug-like compounds, non-drug-like compounds and natural compounds from traditional Chinese medicines. *Journal of Cheminformatics*.

[36] Tian S., Li Y., Wang J. (2013). Drug-likeness analysis of traditional Chinese medicines: 2. Characterization of scaffold architectures for drug-like compounds, non-drug-like compounds, and natural compounds from traditional Chinese medicines. *Journal of Cheminformatics*.

[37] Tian S., Wang J., Li Y., Xu X., Hou T. (2012). Drug-likeness analysis of traditional chinese medicines: prediction of drug-likeness using machine learning approaches. *Molecular Pharmaceutics*.

[38] Xue M., Zhang S., Cai C. (2013). Predicting the drug safety for traditional Chinese medicine through a comparative analysis of withdrawn drugs using pharmacological network. *Evidence-Based Complementary and Alternative Medicine*.

[39] Hopkins A. L. (2008). Network pharmacology: the next paradigm in drug discovery. *Nature Chemical Biology*.

[40] Csermely P., Ágoston V., Pongor S. (2005). The efficiency of multi-target drugs: the network approach might help drug design. *Trends in Pharmacological Sciences*.

[41] Araujo R. P., Doran C., Liotta L. A., Petricoin E. F. (2004). Network-targeted combination therapy: a new concept in cancer treatment. *Drug Discovery Today: Therapeutic Strategies*.

[42] Li S. (2009). Network systems underlying traditional Chinese medicine syndrome and herb formula. *Current Bioinformatics*.

[43] Zhao S., Li S. (2010). Network-based relating pharmacological and genomic spaces for drug target identification. *PLoS ONE*.

[44] Wu X., Jiang R., Zhang M. Q., Li S. (2008). Network-based global inference of human disease genes. *Molecular Systems Biology*.

[45] Zhao S., Li S. (2012). A co-module approach for elucidating drug-disease associations and revealing their molecular basis. *Bioinformatics*.

[46] Gu J., Zhang H., Chen L., Xu S., Yuan G., Xu X. (2011). Drug-target network and polypharmacology studies of a Traditional Chinese Medicine for type II diabetes mellitus. *Computational Biology and Chemistry*.

[47] Gu J., Li Q., Chen L. (2013). Platelet aggregation pathway network-based approach for evaluating compounds efficacy. *Evidence-Based Complementary and Alternative Medicine*.

[48] Zhang X., Gu J., Cao L. (2014). Network pharmacology study on the mechanism of traditional Chinese medicine for upper respiratory tract infection. *Molecular BioSystems*.

[49] Wang C., Zhong Y., Zhang Y. (2016). A network analysis of the Chinese medicine Lianhua-Qingwen formula to identify its main effective components. *Mol. BioSyst.*.

[50] Liu J. L., Pei M., Zheng C. L. (2013). A systems-pharmacology analysis of herbal medicines used in health improvement treatment: predicting potential new drugs and targets. *Evidence-Based Complementary and Alternative Medicine*.

[51] Chen G., Lu C., Zha Q. (2012). A network-based analysis of traditional Chinese medicine cold and hot patterns in rheumatoid arthritis. *Complementary Therapies in Medicine*.

[52] Li Q., Li X., Li C. (2011). A network-based multi-target computational estimation scheme for anticoagulant activities of compounds. *PLoS ONE*.

[53] Tao W., Xu X., Wang X. (2013). Network pharmacology-based prediction of the active ingredients and potential targets of Chinese herbal *Radix Curcumae* formula for application to cardiovascular disease. *Journal of Ethnopharmacology*.

[54] Li J., Lu C., Jiang M. (2012). Traditional chinese medicine-based network pharmacology could lead to new multicompound drug discovery. *Evidence-based Complementary and Alternative Medicine*.

[55] Li S., Zhang B., Jiang D., Wei Y., Zhang N. (2010). Herb network construction and co-module analysis for uncovering the combination rule of traditional Chinese herbal formulae. *BMC Bioinformatics*.

[56] Li S., Zhang B., Zhang N. (2011). Network target for screening synergistic drug combinations with application to traditional Chinese medicine. *BMC Systems Biology*.

[57] Meng H., McClendon C. L., Dai Z. (2016). Discovery of Novel 15-Lipoxygenase Activators to Shift the Human Arachidonic Acid Metabolic Network toward Inflammation Resolution. *Journal of Medicinal Chemistry*.

[58] Wu Y., Zhuo X., Dai Z. (2015). Modeling the mitotic regulatory network identifies highly efficient anti-cancer drug combinations. *Mol. BioSyst.*.

[59] Liang H., Ruan H., Ouyang Q., Lai L. (2016). Herb-target interaction network analysis helps to disclose molecular mechanism of traditional Chinese medicine. *Scientific Reports*.

[60] Yang K., Ma W., Liang H., Ouyang Q., Tang C., Lai L. (2005). Dynamic simulations on the Arachidonic Acid Metabolic Network. *PLoS Computational Biology*.

[61] Yang K., Bai H., Ouyang Q., Lai L., Tang C. (2008). Finding multiple target optimal intervention in disease-related molecular network. *Molecular Systems Biology*.

[62] Wei D., Jiang X., Zhou L. (2008). Discovery of multitarget inhibitors by combining molecular docking with common pharmacophore matching. *Journal of Medicinal Chemistry*.

[63] Chen Z., Wu Y., Liu Y., Yang S., Chen Y., Lai L. (2011). Discovery of dual target inhibitors against cyclooxygenases and leukotriene A4 hydrolyase. *Journal of Medicinal Chemistry*.

[64] Wu Y., He C., Gao Y., He S., Liu Y., Lai L. (2012). Dynamic modeling of human 5-lipoxygenase-inhibitor interactions helps to discover novel inhibitors. *Journal of Medicinal Chemistry*.

[65] Kanehisa M., Goto S. (2000). KEGG: kyoto encyclopedia of genes and genomes. *Nucleic Acids Research*.

